# Autoregulatory loop between TGF-*β*1/miR-411-5p/SPRY4 and MAPK pathway in rhabdomyosarcoma modulates proliferation and differentiation

**DOI:** 10.1038/cddis.2015.225

**Published:** 2015-08-20

**Authors:** M Sun, F Huang, D Yu, Y Zhang, H Xu, L Zhang, L Li, L Dong, L Guo, S Wang

**Affiliations:** 1 Laboratory Animal Research Center, Soochow University School of Medicine, Suzhou 215123, China; 2 Department of Pathology, Soochow University School of Medicine, Suzhou 215123, China; 3 Department of Plastic Surgery, Second Affiliated Hospital, Soochow University, Suzhou 215004, China; 4 Department of Oncology, First Affiliated Hospital, Soochow University, Suzhou 215006, China; 5 Department of Surgery, First Affiliated Hospital, Soochow University, Suzhou 215006, China; 6 Department of Osteology, Second Affiliated Hospital, Soochow University, Suzhou 215004, China

## Abstract

The origin of rhabdomyosarcoma (RMS) remains controversial. However, specific microRNAs (miRNAs) are downregulated in RMS and it is possible that re-expression of these miRNAs may lead to differentiation. Transforming growth factor-*β*1 (TGF-*β*1) is known to block differentiation of RMS. We therefore analyzed miRNA microarrays of RMS cell lines with or without TGF-*β*1 knockdown and identified a novel anti-oncogene miR-411-5p. Re-expression of miR-411-5p inhibited RMS cell proliferation *in vitro* and tumorigenicity *in vivo*. Using a luciferase reporting system and sequence analysis, the potential target of miR-411-5p was identified as sprouty homolog 4 (*SPRY4*), which inhibits protein kinase C*α*-mediated activation of mitogen-activated protein kinases (MAPKs), especially p38MAPK phosphorylation. These results revealed an inverse correlation between TGF-*β*1/SPRY4 and miR-411-5p levels. *SPRY4* small interfering RNA and miR-411-5p both activated p38MAPK phosphorylation and also promoted apoptosis and myogenic differentiation, indicated by increased caspase-3, myosin heavy chain, and myosin expression. *SPRY4* and miR-411 mRNA levels correlated with TGF-*β*1 expression levels in RMS tissues, which was confirmed by immunohistochemical staining for TGF-*β*1, SPRY4, and phosphorylated p38MAPK proteins. Overall, these results indicate that miR-411-5p acts as an RMS differentiation-inducing miRNA prompting p38MAPK activation via directly downregulating *SPRY4*. These results establish an autoregulatory loop between TGF-*β*1/miR-411-5p/SPRY4 and MAPK in RMS, which governs the switch between proliferation and differentiation.

Rhabdomyosarcoma (RMS) is the most common soft tissue sarcoma in pediatric patients and young adults. RMS is typically divided into two histopathologic subgroups: alveolar RMS (ARMS) and embryonal RMS (ERMS). ERMS and ARMS are the most prevalent, accounting for 70 and 20% of RMS cases, respectively.^1^ Most ARMS tumors express fusion proteins combining the DNA-binding domain of either PAX3 or PAX7 transcription factors with the transcriptional-activation domain of FOXO1A.^2, 3^ ERMS accounts for most RMS cases, although its molecular basis remains poorly understood. However, studies in mice and humans have implicated mutations in the p53, Sonic hedgehog and Rb1 in the pathogenesis of ERMS.^4, 5, 6^ In the case of RMS, the tissue of origin closely resembles striated skeletal muscle, with high expression levels of pro-myogenic proteins such as MyoD, myogenin, and desmin. However, despite the expression of these myogenic regulators, RMS is retained at different stages of myogenic differentiation.

Some muscle-specific microRNAs (miRNAs) that act as key regulators of skeletal muscle cell fate determination have recently been shown to be strikingly decreased in both ARMS and ERMS.^7, 8, 9, 10^ Interestingly, re-expression of these specific miRNAs, such as miR-1 and miR-206, impaired the tumorigenic behavior of RMS cells *in vivo*,^8, 9^ raising the possibility that miRNA re-expression may represent an effective differentiation therapy for RMS.^11^ The potential efficacy of differentiation therapy that exploits the regulation of skeletal muscle differentiation by miRNAs in RMS is based on the hypothesis that RMS arises from skeletal muscle cells. These specific miRNAs act as differentiation regulators during the process of muscle differentiation because of their predicted selectivity for genes involved in the myogenic program. However, despite its myogenic phenotype, the origin of RMS remains uncertain; it can arise from salivary glands,^12^ the genitourinary tract,^13^ and the adipocyte lineage.^14^ The above muscle-specific miRNAs, selected by comparing RMS with normal skeletal tissue, can restore the differentiation state of RMS; however, other tissue-specific miRNAs, detected by comparison with other originating tissues, may also be effective. The nature of RMS differentiation blockage is thus not yet fully understood.

Transforming growth factor-*β*1 (TGF-*β*1) is well characterized as a critical modulator of skeletal muscle differentiation.^15, 16^ Previous studies have shown that inhibition of TGF-*β*1 signaling in human RMS promotes myogenic differentiation.^17, 18, 19^ TGF-*β*1 signaling from the cell surface to the nucleus can also be directed through mitogen-activated protein kinases (MAPKs), such as the extracellular signal-regulated kinase 2 (ERK2) or TGF-*β*1/Smad pathways.^20^ Growth-arresting signals can be triggered either by activation of Jun N-terminal kinases (JNKs) or by preventing both ERK and JNK activation, and sarcomeric myosin expression was induced by the ERK inhibitor U0126, but abrogated by the p38MAPK inhibitor SB203580.^21^ These results indicate the existence of a relationship between TGF-*β*1 signaling and p38MAPK inactivation in RMS. However, the causes of sustained ERK activation and p38MAPK inactivation in RMS, and the downstream effectors of MAPKs that elicit specific responses, have not yet been fully identified.

Ongoing studies have established that some miRNAs that act as regulators of differentiation are regulated by TGF-*β*1 signaling, for example, miR-24, which exerts positive effects on myogenic differentiation but is suppressed by TGF-*β*1.^16^ Recent studies have begun to demonstrate that miRNAs are both regulators of and regulated by TGF-*β*1 signaling pathways. Complex interactions between TGF-*β*1 signaling and miR-29, miR-206, and miR-450b-5p are mediated by members of the Smad protein family. miR-206 and miR-29 were shown to attenuate the inhibitory actions of TGF-*β*1 on myogenic differentiation.^22^ miR-450b-5p unites several molecular pathways; it is affected by Smad3 and 4 (but not Smad2) to influence ecto-NOX disulfide-thiol exchanger 2 (ENOX2) and paired box9 (Pax9).^19^ These findings underscore the need to clarify the roles of TGF-*β*1-regulated miRNAs in regulation of the behavior of RMS.

In this study, we investigated the involvement of the novel TGF-*β*1-suppressed anti-oncogenic miRNA miR-411-5p in RMS. miR-411-5p was recently reported to be upregulated in facioscapulohumeral muscular dystrophy and to suppress myogenic factors,^23^ while being decreased in RMS. We investigated the targets and mechanisms of miR-411-5p in RMS using mimics and inhibitors, and demonstrated the involvement of sprouty homolog 4 (SPRY4) and MAPK activation, thus identifying potential novel targets for RMS differentiation therapy.

## Results

### miR-411-5p expression negatively regulated by TGF-*β*1 in RMS

**Figure 1:**
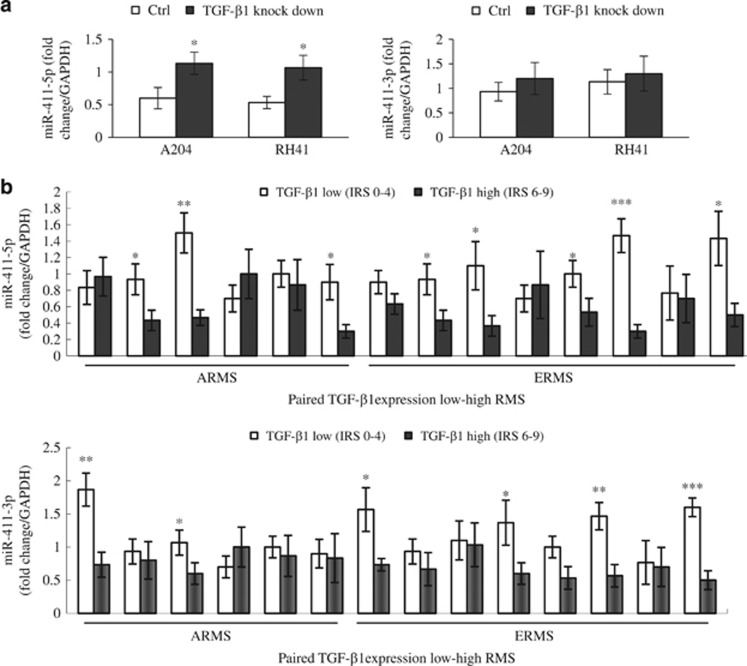
miR-411 expression negatively with TGF-*β*1 in human RMS cell lines and tissues. (**a**) The bar graphs indicate the results of RT-PCR analysis of the miR-411-3p/5p using RNA isolated from TGF-*β*1-knockdown and control RMS cell lines (ERMS: A204; ARMS: RH41). (**b**) RT-PCR analysis of the miR-411-3p/5p using RNA isolated from six paired low-/high-TGF-*β*1-expressing ARMS and eight paired ERMS tissue. The IRS of TGF-*β*1 is explained in Materials and Methods. Each assay was conducted at least three times independently. Error bars indicate S.D. *P<0.05; **P<0.01; ***P<0.005

We explored the effects of TGF-*β*1 on miRNA suppression and the behavior of RMS based on the differentially expressed miRNA profiles in TGF-*β*1-knockdown cell lines (RD, SMS-CTR, and RH28) using comprehensive locked nucleic acid (LNA) microarray analyses (Gene Expression Omnibus series accession number GSE40843). miR-4275, miR-411, miR-493*, miR-450b-5p, and miR-4298 were all significantly upregulated in TGF-*β*1-knockdown RMS cell lines *versus* controls as previously reported.^19^ We also examined other TGF-*β*1-knockdown RMS cell lines (A204 and RH41) and found that miR-411-5p and miR-411-3p expression levels were significantly increased in four and two of these four lines (ERMS: A204; ARMS: RH41), respectively (see Figure 1a and Supplementary Figure S1 and Supplementary Figure S1 of Sun *et al.*^19^).

We examined the miR-411 cluster by reverse transcription-PCR (RT-PCR) using total RNA isolated from six low- and high-TGF-*β*1-expressing ARMS samples and eight paired ERMS samples, based on immunoreactive score (IRS) of TGF-*β*1 protein expression obtained as described previously.^17^ As anticipated, miR-411-5p expression differed significantly between low- and high-TGF-*β*1-expressing ARMS and ERMS tissues in three of six and five of eight lines, respectively, whereas miR-411-3p expression differed significantly in two of six and four of eight, respectively (Figure 1b). These results suggest that TGF-*β*1 had a greater regulatory effect on miR-411-5p than miR-411-3p in RMS. We therefore selected miR-411-5p for further study.

### miR-411-5p inhibited proliferation of RMS *in vitro* and *in vivo*

**Figure 2:**
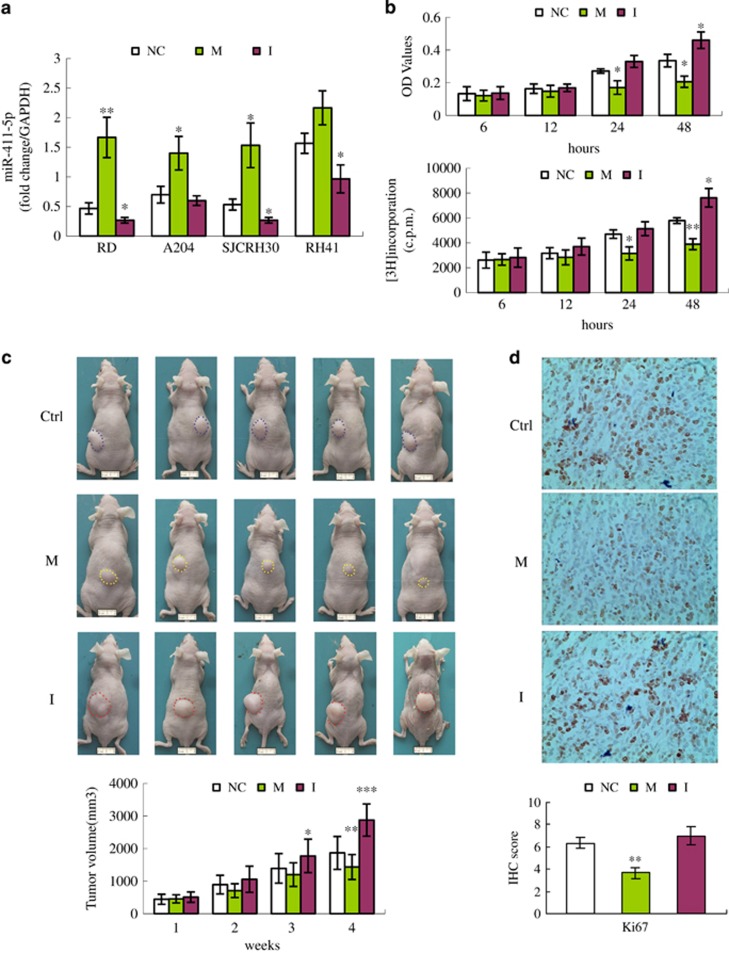
Effect of miR-411-5p on proliferation of RMS cells *in vitro* and *in vivo*. (**a**) RT-PCR analysis of miR-411-5p levels in RMS cell lines (RD, SMC-CTR, RH28, and RH3) treated with miR-411-5p mimics (M), inhibitors (I), and negative control (NC) (see Materials and Methods). (**b**) Growth of RD cells transfected with miR-411-5p-M, -I, or NC according to MTT assay and [^3^H]thymidine incorporation. (**c**) Tumor volumes of RD xenografts in nude mice injected with miR-411-5p-M, -I, or control (Ctrl) twice weekly for 4 weeks. A total of 60 tumors were analyzed (20 tumors per group). Images show representative tumor spheres (scale bar=20 mm). (**d**) Immunohistochemical staining of RMS xenograft sections derived from RD cells injected with miR-411-5p-M, -I, or Ctrl oligonucleotides for 4 weeks. Ki67-specific antibody was used as the marker for cell proliferation. Original magnification, × 400. All values given as mean±S.D. from three independent experiments. *P<0.05; **P<0.01; ***P<0.005

To determine the effect of miR-411-5p on RMS behavior, we transiently transfected miR-411-5p mimics (miR-411-5p-M) and inhibitors (miR-411-5p-I) into RD and SJCRH30 cells *in vitro*, and explored RMS cell proliferation by the [3H]thymidine incorporation and MTT (3-(4,5-dimethylthiazol-2-yl)-2,5-diphenyltetrazolium bromide) assay. The expression of miR-411-5p was confirmed by real-time RT-PCR analysis (Figure 2a) after confirmation that >80% of cells were transfected with a synthetic oligonucleotide marked with carboxyfluorescein (FAM) (data not shown). The optical density (OD) and [^3^H]thymidine incorporation were reduced in RD cells by treatment with miR-411-5p-M, in a time-dependent manner. Significant differences in proliferation of RD cells were observed after 24 h of treatment (Figure 2b). Similar results were found in the RMS cell line SJCRH30 (Supplementary Figure S2). Although these cells are derived from different backgrounds, they bear common genetic abnormalities such as t(2;13)(q35;q14)^24^ and p53 loss.^25^ miR-411-5p was decreased in most RMS cell lines and specimens, and this common genetic abnormality may thus have contributed to the similar results in both cell lines.

To determine whether the observed growth arrest of RMS *in vitro* was associated with repression *in vivo*, we quantified tumorigenicity using a nude mouse model (see Materials and Methods). As shown in Figure 2c, there was a significant difference between mice treated with miR-411-5p-I (GroupI) and the control group after 3 weeks (*P*<0.05), and the difference was even greater after 4 weeks (*P*<0.005). Tumor volume in the miR-411-5p-I-treated group increased 1.5-fold compared with the control group. miR-411-5p-M-treated mice (Group M) showed a significant decrease in tumor volume after 4 weeks (*P*<0.01). These results suggest that miR-411-5p had an inhibitory effect on RD cell proliferation *in vivo*. This was further confirmed by analysis of Ki67 expression (*P*<0.01), as shown in Figure 2d.

### SPRY4 is a direct target of miR-411-5p

**Figure 3:**
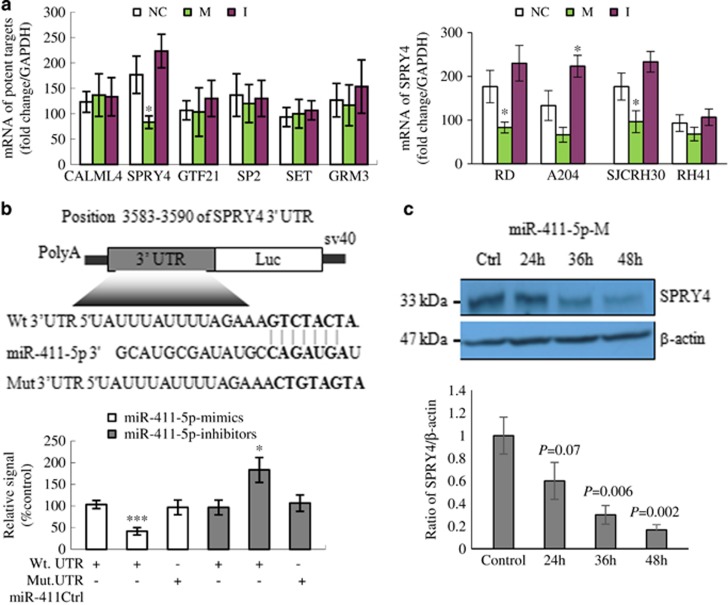
SPRY4 is the target for miR-411-5p. (**a**) mRNA levels of six potential targets of miR-411-5p (*CALML4*, *SPRY4*, *GTF21*, *SP2*, *SET*, and *GRM3*) in RD cells transfected with miR-411-5p mimic (M), miR-411-5p inhibitor (I), and negative control (NC). *SPRY4* was the most significantly downregulated by miR-411-5p-M in RD cells. The results were confirmed in other RMS cells (ERMS: A204; ARMS: SJCRH30 and RH41). (**b**) Diagram of *SPRY4* 3′-UTR-containing reporter constructs and results of luciferase assays (see Materials and Methods). RD cells were transfected with Wt or mutated (Mut) reporter constructs and miR-411-5p-M or -I. Luciferase assays confirmed that miR-411-5p bound to Wt 3′-UTR sequences of *SPRY4* in RD cells. Co-transfection of miR-411-5p-M significantly reduced luciferase levels, whereas miR-411-5p-I increased luciferase levels. The reporter construct mutated at the specific target site was unaffected by simultaneous transfection. (**c**) Immunoblotting of total protein lysates from RD cells treated with miR-411-5p-M using SPRY4 antibodies. *β*-Actin was used as a loading control. SPRY4 protein levels were downregulated in RD cells in a time-dependent manner by miR-411-5p-M

Six overlapping potential targets of miR-411-5p were predicted by three different bioinformatic algorithms (see Materials and Methods): calmodulin-like 4 (*CALML4*), *SPRY4*, general transcription factor 21 (*GTF21*), transcription factor (*SP2*), SET nuclear oncogene (*SET*), and glutamate receptor metabotropic 3 (*GRM3*). Among these candidates, only *SPRY4* was significantly repressed by miR-411-5p-M and augmented by miR-411-5p-I in RD cells (Figure 3a). This result was confirmed independently in two of three other RMS cell lines (ERMS: A204; ARMS: SJCRH30 and RH41). *SPRY4* miRNA expression was increased in miR-411-5p-I-treated and decreased in miR-411-5p-M-treated RH41 cells, although the difference was not significant (data not shown).

To determine whether the 3′-untranslated region (UTR) of *SPRY4* was the functional target of miR-411-5p, we examined the reporter activity of the wild-type (Wt) 3′-UTR sequences of *SPRY4*. As shown in Figure 3b, *SPRY4* signals were decreased by co-transfection with miR-411-5p-M in RD cells and increased by co-transfection with miR-411-5p-I. However, the activity of a reporter construct mutated at the specific target site was unaffected by simultaneous transfection. These results support the targeting relationship between miR-411-5p and *SPRY4* at the gene level. We also confirmed that miR-411-5p-M downregulated SPRY4 at the protein level in RD cells and SPRY4 protein was almost undetectable at 36 h after treatment (Figure 3c). In addition, we confirmed the 3′-UTR of SPRY4 as the functional target of miR-411-5p and miR-411-5p-M in the downregulation of SPRY4 at the protein level in the SJCRH30 ARMS cell line (Supplementary Figure S3).

### SPRY4 suppresses PKC*α*-mediated MAPK activation

**Figure 4:**
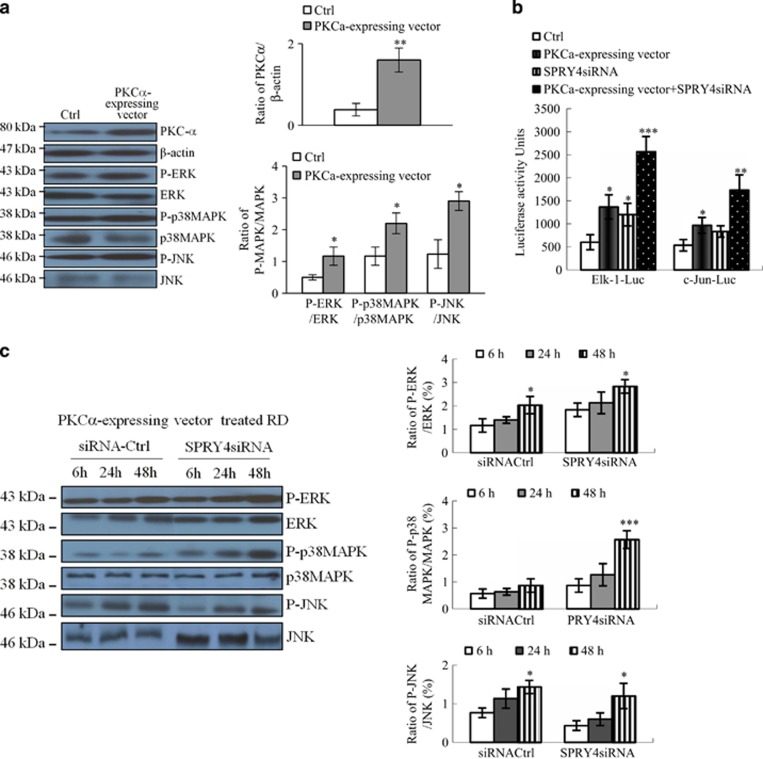
Effect of SPRY4 on PKC*α*-mediated MAPK activation. (**a**) Western blot analysis of PKC*α*, P-ERK, P-JNK, and P-p38MAPK in lysates of RD cells transfected with PKC*α* cDNA expression constructs and controls. (**b**) Activated Elk-1 and c-Jun detected by luciferase assay. RD cells co-transfected with Elk-1 and c-Jun activator plasmid, and GAL4-luc reporter plasmid together with PKC*α*-expressing vector; empty vector (Ctrl) were untreated or treated with *SPRY4*siRNA or *SPRY4*siRNA plus PKC*α*-expressing vector. (**c**) Immunoblot analysis of ERK, p38MAPK, and JNK phosphorylation in PKC*α*-expressing-vector-treated RD cells simultaneously treated with either *SPRY4*siRNA or control siRNA (Ctrl) for 6, 24, and 48 h. Each assay (**a**–**c**) was conducted at least three times independently. Error bars indicate S.D. *P<0.05; **P<0.01; ***P<0.005

SPRY4 has been shown to have a negative regulatory effect on protein kinase C (PKC) activation induced by vascular endothelial growth factor-A^26^ and PKC*α*-mediated MAPK activation leads to growth arrest of RMS.^21^ This suggests that SPRY4 may be involved in the regulation of RMS growth by suppressing PKC activation. To determine the effect of PKC*α* on MAPK kinase phosphorylation, we analyzed total lysate from RD cells treated with either control vector or constitutively active PKC*α*-expressing vector, using antibodies against phosphorylated, active forms of MAPKs. As shown in Figure 4a, PKC*α* expression was increased 4.2-fold in PKC*α*-expressing vector-treated RD cells, associated with significant increases in ratio of phosphorylated (P)-ERK to ERK (2.4-fold), phosphorylated p-38 (P-p38MAPK) to p38MAPK (1.8-fold), and P-JNK to JNK(2.4-fold) compared with the ratio of P-MAPK to MAPK in controls. To assess the role of PKC*α* in MAPK activation further, we used a luciferase reporting system (pFA-Elk-1 or pFA-c-Jun) and assayed activated Elk-1 and c-Jun in RD cells co-transfected with PKC*α*-expressing vector with activator plasmid (see Materials and Methods) and pFR-Luc (reporter plasmid), with or without *SPRY4* small interfering RNA (siRNA). As shown in Figure 4b, the luciferase activities of Elk-1 and c-Jun were increased 2.3- and 1.8-fold, respectively, by the PKC*α*-expressing vector compared with the control. The luciferase activities of Elk-1 and c-Jun were also induced by *SPRY4*siRNA in both PKC*α*-expressing vector-transfected (4.3- and 3.3-fold, respectively) and control (2- and 1.6-fold, respectively) cells. These results confirmed that MAPK kinase phosphorylation was activated by PKC*α*. However, the phosphorylation-inhibitory effect of SPRY4 on the PKC*α*-dependent MAPK pathway needs to be explored further.

Next, we examined the time courses of ERK, p38MAPK, and JNK phosphorylation in PKC*α*-expressing vector-treated RD cells treated with *SPRY4*siRNA or control siRNA (see Materials and Methods). As shown in Figure 4c, ERK and JNK phosphorylation were significantly increased over 48 h in control (1.7-fold and 1.9-fold, respectively) and *SPRY4*siRNA-treated (1.5-fold and 2.1-fold, respectively) cells. Interestingly, p38MAPK phosphorylation was not significantly increased in control cells, but was increased 3.0-fold in *SPRY4*siRNA-treated cells. Taken together, these results indicate that *SPRY4* had an inhibitory effect on PKC*α*-dependent MAPK kinase phosphorylation, in particular p38MAPK.

### SPRY4 suppresses p38MAPK activation contributing to RMS differentiation blockage

**Figure 5:**
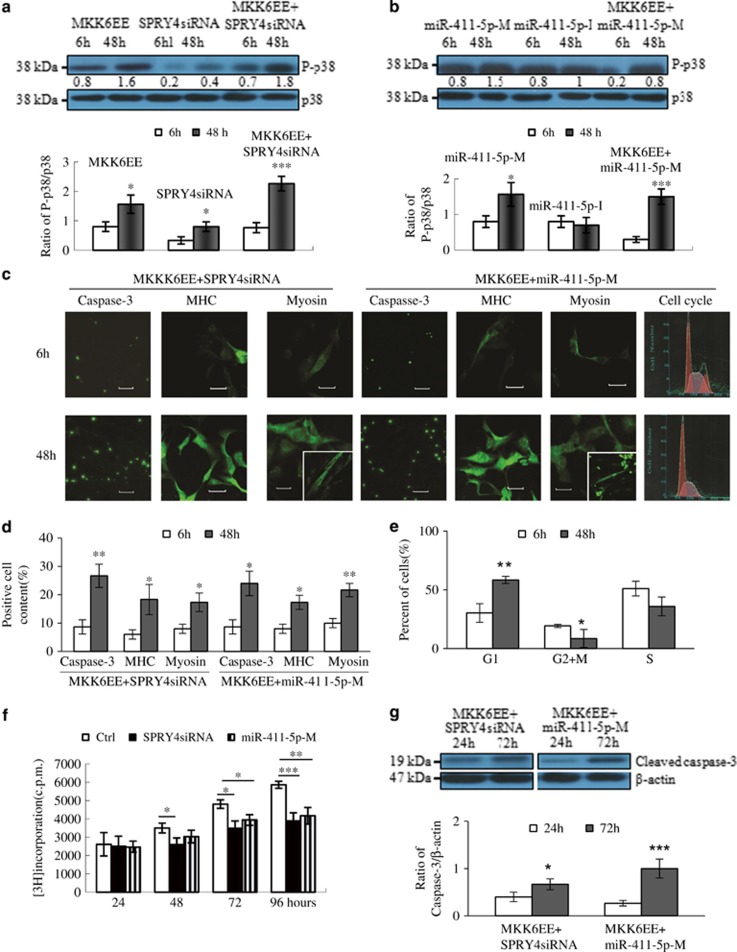
miR-411-5p activate p38MAPK pathway by targeting SPRY4-induced terminal differentiation of RMS. (**a**) Western blot analysis of p38MAPK activation (P-p38MAPK: p-38) in RMS cells treated with HA-tagged MKK6EE protein-expressing pcDNA3, *SPRY4*siRNA, or both. (**b**) Western blot analysis of p38MAPK activation (P-p38MAPK:p-38) in RMS cells treated with miR-411-5p-M, miR-411-5p-I, and MKK6EE protein-expressing pcDNA3 plus miR-411-5p-M. (**c**) Terminal differentiation of RD cells after treatment with *SPRY4*siRNA or miR-411-5p-M for 6 and 48 h. Cells stained with apoptosis marker (caspase-3); cell cycle was analyzed using propidium iodide (PI) and showed changed morphology (MHC and myosin) after activation of p38MAPK caused by *SPRY4*siRNA or miR-411-5p-M. Scale bars: panel (Caspase-3)=100 *μ*m, panel (MHC and Myosin)=20 *μ*m. (**d**) Quantitation of terminal differentiation of RD cells after treatment with *SPRY4*siRNA or miR-411-5p-M for 6 and 48 h. (**e**) Graphical representation of cell cycle phase proportions in RD cells after treatment with miR-411-5p-M for 6 and 48 h. (**f**) Proliferation of RD cells was evaluated by [^3^H] thymidine incorporation assay after treatment with *SPRY4*siRNA or miR-411-5p-M for 4 days. The amount of [^3^H] thymidine incorporated was measured with a liquid scintillation counter. The OD at 557 nm was determined using a microplate reader. (**g**) Western blot analysis of cleaved caspase-3 in RMS cells treated with *SPRY4*siRNA or miR-411-5p-M for 24 and 72 h. Each assay (**a**–**g**) was conducted at least three times independently. Error bars indicate S.D. *P<0.05; **P<0.01; ***P<0.005

Constitutive p38MAPK activation has been shown to cause growth arrest and terminal differentiation in RMS cells.^27^ We further investigated the role of *SPRY4* in inhibiting p38MAPK activation by determining whether p38MAPK deliberately activated by ectopic expression of the constitutive active form of MAPK kinase 6 (MKK6EE) could bypass the inhibitory effect of *SPRY4*. Increased tumor growth is known to be generally correlated with reduced differentiation. Caspase-3 has been recognized as a main effector caspase in the apoptotic cascade. We analyzed the numbers of caspase-3 (green nuclear staining), myosin heavy chain (MHC)-, and myosin-positive cells (green cytoplasm staining). As shown in Figure 5a, ectopic expression of MKK6EE or *SPRY4* knockdown alone (48 h) significantly activated p38MAPK phosphorylation, but failed to promote cell cycle arrest (caspase-3) and efficient morphological conversion (proportions of myotube-like and multinucleated myofiber-like cells) (data not shown). However, co-transfection of MKK6EE and *SPRY4*siRNA synergistically activated p38MAPK phosphorylation and also promoted apoptosis and myogenic differentiation, indicated by increased numbers of caspase-3-, MHC-, and Myosin-positive cells, and myotube-like cells (Figures 5c and d).

To determine whether miR-411-5p could promote p38MAPK phosphorylation, we examined p38MAPK in miR-411-5p-M- or miR-411-5p-I-treated RD cells and RD cells co-transfected with MKK6EE and miR-411-5p-M. As expected, miR-411-5p-M alone (48 h) activated p38MAPK phosphorylation (twofold) (Figure 5b). However, co-transfection of MKK6EE with miR-411-5p-M obviously induced p38MAPK phosphorylation (fivefold), promoted caspase-3 and MHC expression, and converted a proportion of RD cells to myofiber-like cells (Figures 5c and d), consistent with the effects of co-transfecting MKK6EE and *SPRY4*siRNA. The results of flow cytometry analysis of the cell cycle are shown in Figures 5c and e. Following co-transfection of MKK6EE with miR-411-5p-M, cells accumulated in G1 and were relatively depleted in the S and G2/M phases (Figures 5c and e).

To confirm the inhibitory roles of miR-411-5p and *SPRY4*siRNA on RMS proliferation, we examined [^3^H]thymidine incorporation and caspase-3 protein by RMS cells treated with miR-411-5p-M and *SPRY4*siRNA, respectively. After 48 h of treatment, *SPRY4*siRNA significantly decreased [^3^H]thymidine incorporation in RMS cells. After prolonged treatment (1–4 days), the decreases in [^3^H]thymidine incorporation in RMS cells induced by *SPRY*siRNA and miR-411-5p-m (Figure 5f) were consistent with the results of caspase-3 protein analysis (Figure 5g). Overall, these results suggest that *SPRY4*siRNA and miR-411-5p were both able to arrest the growth of RMS by activating p38MAPK phosphorylation.

### Relationships between TGF-*β*1, SPRY4, and P-p38MAPK expression in RMS tissues and clinical pathology

**Figure 6:**
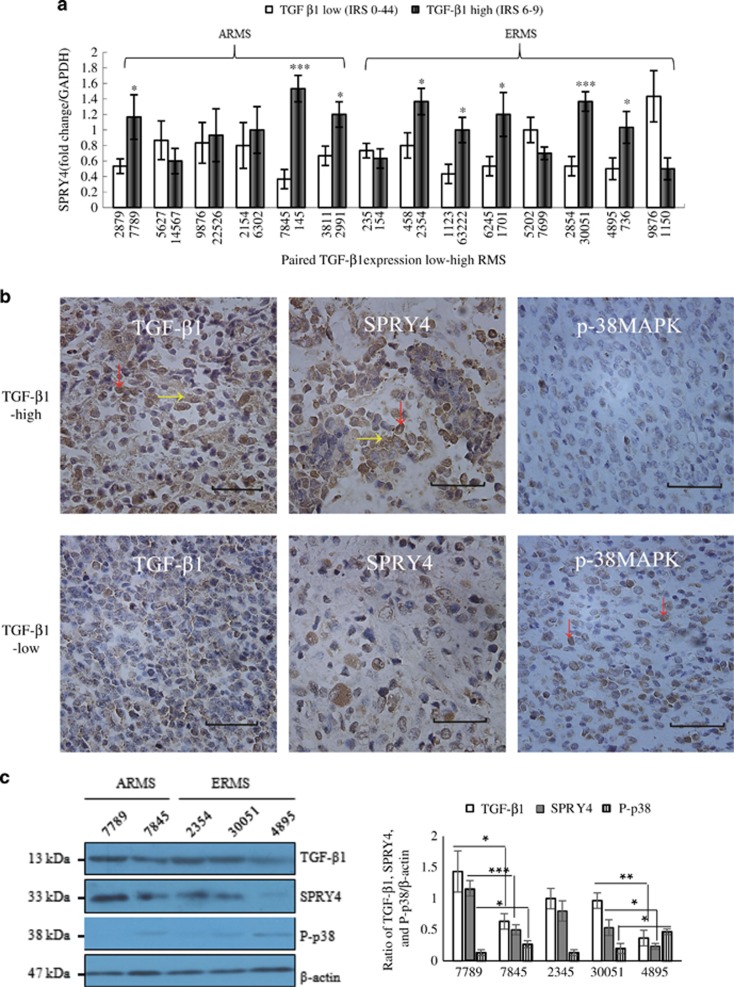
Relationships between TGF-*β*1, SPRY4, and P-p38MAPK expression in RMS. (**a)** *SPRY4* expression was determined by RT-PCR in six paired high- (IRS 6–9) and low-TGF-*β*1 (IRS 0–4) ARMS and eight paired ERMS tissues. Numbers indicate tissue numbers. Bars indicate mRNA expression levels normalized to glyceraldehyde 3-phosphate dehydrogenase (GAPDH)±S.D. (**b**) Immunohistochemical staining for TGF-*β*1, SPRY4, and P-p38MAPK in RMS tissue. Representative immunohistochemical staining of TGF-*β*1-high ERMS (tissue number 2354) and TGF-*β*1-low ERMS (tissue number 458). Original magnification, × 400. Scale bar=50 *μ*m. For quantitative analysis of SPRY4 expression, see Supplementary Figure 4. (**c**) Western blot analysis of TGF-*β*1, SPRY4, and P-p38MAPK in tissue lysates from randomly sampled ARMS (7789 and 7845) and ERMS (2345, 30051, and 4985) tissues. *β*-Actin was used as a loading control. Each assay was conducted at least three times independently. Error bars indicate S.D. *P<0.05; **P<0.01; ***P<0.005

Based on our previously optimized scoring system for TGF-*β*1 expression in paraffin-embedded RMS tissues,^17^ we paired low- (IRS 0–4) and high- (IRS 6–9) TGF-*β*1-expressing RMS tissues and analyzed the relationship between SPRY4 and P-p38MAPK expression levels and TGF-*β*1 expression. Analysis of *SPRY4* mRNA by RT-PCR showed that high *SPRY4* mRNA expression levels were associated with high TGF-*β*1 expression (8/16). As shown in Figure 6a, three of six paired ARMS and five of eight paired ERMS tissues showed significant expression of *SPRY4* (*P*<0.05), indicating a positive correlation between *SPRY4* and TGF-*β*1 at the mRNA level.

We then used immunohistochemistry to investigate TGF-*β*1, SPRY4, and P-p38MAPK protein expression levels in RMS tissues. As shown in Figure 6b, TGF-*β*1 was localized in the cytoplasm and nucleus in RMS tissues, with some diffuse and local staining. In contrast, P-p38MAPK was distributed within the whole nucleus and SPRY4 was detected predominantly in the nucleus, with lower levels in the cytoplasm. SPRY4 was significantly overexpressed in most high-TGF-*β* tissues, whereas P-p38MAPK showed the opposite tendency with P-p38MAPK being overexpressed largely in low-TGF-*β* tissues (Supplementary Figure S4). These results were further confirmed by western blotting (Figure 6c).

We examined the correlations between TGF-*β*1, SPRY4, and P-p38MAPK expression levels and clinical pathology (Table 1). Regarding histological subtypes, high IRS (6~9) of TGF-*β*1 in ERMS, ARMS, and PRMS were 72.9, 66.7, and 83.3%, respectively, whereas SPRY4 levels were similar at 79.2, 61.1, and 83.3%, respectively. In contrast, the high IRS of P-p38MAPK were lower, at 25.0, 27.8, and 16.7%, respectively. Further analysis of TGF-*β*1, SPRY4, and P-p38MAPK proteins in relation to the degree of differentiation showed that both TGF-*β*1 and SPRY4 levels were significantly increased in RMS with low grades of differentiation compared with RMS with high grades of differentiation. In contrast, P-p38MAPK protein expression decreased with increasing grade of differentiation. Among 20 cases with TGF-*β*1-low RMS, 17 showed low SPRY4 expression (85%), whereas 15 showed high P-p38MAPK expression (75%). However, among 52 cases with TGF-*β*1-high RMS, 40 showed high SPRY4 expression (77%), but only 8 showed high P-p38MAPK expression (15%). In addition, high expression levels of TGF-*β*1, SPRY4, and P-p38MAPK were found in 77.4, 83.9, and 9.7% of RMS patients with relapse or metastasis, respectively, compared with 36.6, 41.5, and 24.4% of patients with recurrence-free survival. This suggests that overexpression of TGF-*β*1 or SPRY4 was indicative of a poor prognosis in patients with RMS, whereas overexpression of P-p38MAPK was associated with a better prognosis.

## Discussion

Rapid progress in our knowledge of miRNAs has opened up new diagnostic and therapeutic opportunities.^28^ Some miRNAs act as oncogenes, whereas other miRNAs that are downregulated in cancer are commonly anti-oncogenes. Previous studies have demonstrated that normalization of miRNA expression, mostly of tissue-specific miRNAs, could be used as differentiation therapy in RMS.^11^ Further investigation of the specific miRNAs downregulated in RMS may therefore identify promising therapeutic options. However, the origin of RMS is uncertain and identifying specifically downregulated miRNAs by comparing RMS tissues with the presumed tissue of origin is unreliable. The present study worked on the hypothesis that TGF-*β*1-suppressed miRNAs might represent RMS-specific miRNAs, based on the evidence that TGF-*β*1 signaling blocked differentiation of RMS.^17, 18^ We recently verified this hypothesis by demonstrating that restoration of miR-450b-5p caused cell growth arrest and enhanced MyoD signaling both *in vitro* and *in vivo* xenotransplant experiments.^19^

TGF-*β*1 signaling has been reported to promote metastasis and the invasive potential of cancer cells through various miRNAs, for example, through modulating the biosynthesis of oncogenic miRNAs such as miR-21,^29^ miR-155,^30^ and miR-181b.^31^ In the present study, we performed a genome-wide analysis of TGF-*β*1-regulated miRNA expression in RMS, with a focus on miRNAs suppressed by TGF-*β*1, which may have potential for miRNA re-expression therapy. In addition to miR-450b-5p, the miR-411 cluster represents another set of TGF-*β*1-suppressed miRNAs in RMS. In general, correction of multiple testing in the identification of miRNA should be taken into account, owing to large number of tests. However, this study used *P*<0.05 as the cutoff to select the suggestive miRNA for further validation for the following reasons: (1) the relax cutoff will increase our changes to select the potential significant miRNAs; (2) another selection (fold change >1.5) cutoff was adopted to decrease the likelihood of identifying an miRNA as significant by chance; (3) more importantly, the selection of miRNA based on the array data is the first step and we performed further RT-PCR to quantify the expression miR-411 cluster and validated the differential expression of miR-411 cluster. We demonstrated that both miR-411-3p and miR-411-5p were significantly differentially expressed in various RMS cells and tissues. We chose to concentrate on miR-411-5p, because it showed wider differential expression in RMS than in miR-411-3p. However, the negative relationship between TGF-*β*1 and miR-411-3p was further confirmed by treatment with 5 ng/ml TGF-*β*1 in RD cells (data not shown).

miR-411 is located in 14q32.31 and was recently reported to be upregulated in facioscapulohumeral muscular dystrophy and to suppress myogenic factors.^23^ However, miR-411-5p showed the converse properties in the present study; treatment of RD cells with miR-411-5p-M inhibited proliferation and induced terminal differentiation. The results of the current and previous studies demonstrate that TGF-*β*1 controls RMS differentiation through different miRNAs. miR-450b-5p was suppressed by TGF-*β*1 through Smad3 and Smad4, resulting in overexpression of the target oncogenes ENOX2 and Pax9.^19^ We explored the biological function of miR-411-5p by examining six potential targets, based on the predictions of three different bioinformatic algorithms, and identified *SPRY4* as the most likely target gene of miR-411-5p.

SPRY proteins have major roles in regulating tubular morphogenesis, such as angiogenesis, as well as in placenta, kidney, and lung development.^32, 33, 34^ There are four SPRY orthologs (SPRY1–4) in mammals. SPRY4 expression occurs in various mammalian embryonic tissues, including the brain, heart, muscle, and gut.^35, 36^ SPRY has been reported to be repressed in some cancers and these proteins are thus considered to be tumor suppressors.^37^ However, *SPRY4* was overexpressed in RMS (Supplementary Figure S4).

SPRY4 has been reported as a negative regulator of PKC activation, by inhibiting phosphatidylinositol 4,5-biphosphate hydrolysis,^26^ whereas PKC*α*-mediated MAPK activation is known to promote differentiation of RMS.^21^ We therefore speculated that overexpression of *SPRY4* may contribute to the differentiation blockage in RMS through regulation of MAPK activation. *SPRY4*siRNA promoted phosphorylated ERK, p38MAPK, and JNK in PKC*α*-expressing vector-treated RD cells, suggesting that *SPRY4* has an inhibitory role in MAPK activation. The p38MAPK family comprises several isoforms (p38*α*, p38*β*, p38*γ*, and p38*δ*) with high sequence homology. In this study, we selected p38*α*, which is 75% identical to p38*β* and shows 62 and 61% protein-sequence identities with p38*γ* and p38*δ*, respectively. We further demonstrated that SPRY4 particularly inhibited p38MAPK kinase phosphorylation, as confirmed by ectopic expression of the constitutive active form of MKK6EE. These results support a role for *SPRY4* in the inhibition of MAPK activation.

Previous studies showed that deregulated ERK signaling^38^ and deficient activation of the p38MAPK pathway^21, 27^ contributed to the differentiation blockage of RMS. However, their functional interactions in RMS remain to be investigated. The modulatory function of SPRY proteins on the MAPK signal pathway has been extensively studied. Although they are known as negative regulators of growth-factor-induced ERK activation,^39, 40, 41^ the function of SPRY on different downstream MAPK pathways activated by different stimuli has seldom been reported. Our results provide the first evidence for the role of SPRY in inhibiting activation of the p38MAPK MAPK pathway and on the progression of RMS.

**Figure 7:**
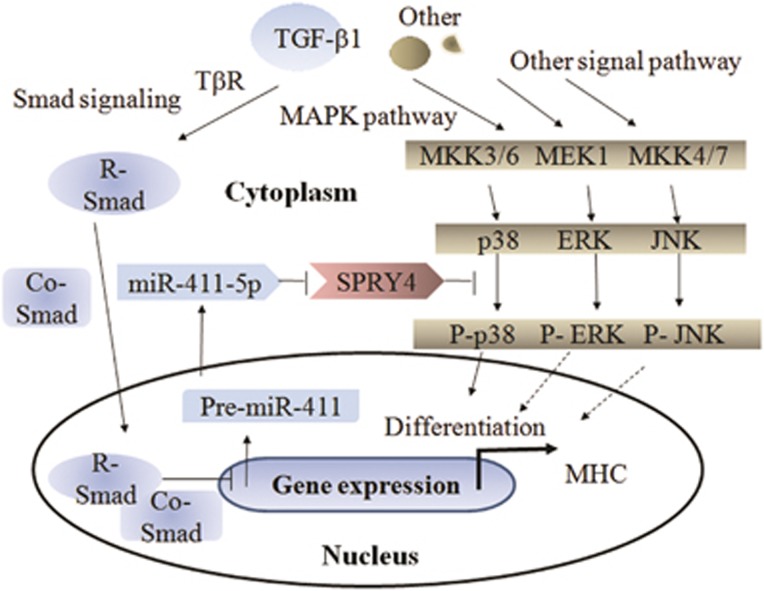
A proposed model of the differentiation blockage by autoregulatory loop between TGF-*β*1/miR-411-5p/SPRY4 and MAPK pathway in RMS. Autocrine TGF-*β*1 suppresses expression of miR-411-5p, resulting in overexpression of its target gene *SPRY4*, which in turn inactivates the MAPK pathway, especially p38MAPK, and contributes to the differentiation blockage of RMS. TGF-*β*1, transforming growth factor-*β*1; R-Smad, restricted smad; Co-Smad, common smad; SPRY4, sprouty homolog 4; MAPK, mitogen-activated protein kinase; MKK, MAPK kinase; ERK, extracellular signal-regulated kinase; JNK, Jun N-terminal kinases; P-p38MAPK, phosphorylated p-38; MHC, myosin heavy chain

Based on an analysis of clinical data, we established a negative correlation between SPRY4 and P-p38MAPK. The expression levels of both TGF-*β*1 and SPRY4 were higher in less well-differentiated RMS and their overexpression was indicative of a poor prognosis; in contrast, P-p38MAPK had the opposite effect. The evidence suggests the existence of an autoregulatory loop between TGF-*β*1/miR-411-5p/SPRY4 and MAPK pathway in RMS (Figure 7), showing that autocrine TGF-*β*1 suppressed the expression of miR-411-5p, resulting in overexpression of the target gene *SPRY4*, which in turn inactivated the MAPK pathway (especially p38MAPK) and contributed to differentiation blockage of RMS, thereby providing a paradigm for an autoregulatory loop between TGF-*β*1/miR-411-5p/SPRY4 and the MAPK pathway in RMS.

## Materials and Methods

### Human cell lines and tissue sampling

The ERMS cell lines RD (ATCC CCL-136) and A204 (ATCC HTB-82), and ARMS cell lines SJCRH30 (ATCC CRL-2061) and RH41 (DSMZ ACC592) were maintained in DMEM. The differentiation medium comprised serum-free DMEM with 10 mg/ml insulin and 10 mg/ml transferring.^42, 43^ RMS tissue was obtained from 72 patients with RMS. Initial biopsy or resection specimens and post-chemotherapy biopsy specimens from residual, recurrent, or metastatic tumor sites were obtained from the Affiliated Hospitals of Soochow University and West China Hospital of Sichuan University. All procedures were carried out in accordance with the recommendations of the Institutional Review Board.

### Construction of TGF-*β*1-knockdown cell lines

The procedure for TGF-*β*1 gene silencing has been described previously.^17^ Briefly, TGF-*β*1-specific siRNAs were designed using Ambion's Target Finder (http://www.ambion.com/techlib/misc/siRNA_finder.html) and synthesized according to the method of Sangon (Shanghai, China). The oligonucleotides were then ligated into the mammalian expression vector pSUPER gfp-neo (VEC-PBS-0006; OligoEngine, Seattle, WA, USA) at the *Bgl*II and *Hind*III cloning sites.

We also established a control-pSUPER gfp-neo vector by changing the *TGF-β1*siRNA sequence. A neomycin-resistance cassette was cloned into the pSUPER vector and transfected into RMS cell lines using Effectene Transfection Reagent (Qiagen, Hilden, Germany). The efficiency of gene silencing was tested by western blot analysis of TGF-*β*1 proteins^17^ and the conventional TGF-*β*1 target Smad7 (Supplementary Figure S5).

### Microarrays and data analysis

Total RNA was isolated from TGF-*β*1-knockdown RMS cell lines (RD, SMS-CTR, and RH28) using TRIzol reagent (Invitrogen, San Diego, CA, USA). RNA was quantitated using a NanoDrop 1000 spectrophotometer (Nanodrop Technologies, Wilmington, DE, USA). miRCURY LNA Array (v.16.0) (Exiqon, Vedbaek, Denmark) was hybridized with 1 *μ*g RNA samples and labeled using the miRCURY Hy3/Hy5 Power Labeling Kit (Exiqon). The slides were scanned using an Axon GenePix 4000B microarray scanner (Axon Instruments, Foster City, CA, USA) and the scanned images were then imported into GenePix Pro 6.0 software (Axon) for grid alignment and data extraction. Hierarchical clustering was performed to determine distinguishable miRNA expression profiles among the samples. Expressed data were normalized using median normalization. Based on the normalized intensities, Student's *t*-test was performed to compare the expression signals between two conditions. Fold change, the ratio of normalized mean intensities between two conditions, was also used to measure the differential expression of miRNA. We adopted two criteria (fold change >1.5, *P*<0.05) to select the miRNAs for the further RT-PCR validation.

### Western blotting and real-time PCR

Cells or tissues were collected and lysed in TNES buffer (10 mm Tris, pH 8.0; 150 mm NaCl; 2 mm EDTA; 0.5% SDS). Equal 25 *μ*g aliquots of proteins were electrophoresed on 8% SDS-polyacrylamide gels and electrotransferred onto polyvinylidine difluoride membranes. The blots were blocked with Tris-buffered saline containing 5% non-fat milk for 1 h. The membranes were incubated with specific antibodies: 1 : 1000 anti-TGF-*β*1, anti-Smad7, anti-SPRY4, anti-PKC*α*, anti-ERK, anti-JNK, and anti-p38MAPK (Santa Cruz Biotechnology, Santa Cruz, CA, USA); and 1 : 1000 anti-p-ERK, anti-p-JNK, and anti-P-p38MAPK (New England BioLabs, Beverly, MA, USA) for 3 h at room temperature, all of which recognize the activated forms of these kinases. Proteins were visualized with peroxidase-conjugated secondary antibodies at 1 : 2000 for 1 h, using an enhanced chemiluminescence detection system (Santa Cruz). Total RNA was collected from RMS cell lines and frozen tissues according to the manufacturer's recommendations. Mature miRNA analysis was performed using TaqMan miRNA assays (Takara Shuzo, Kyoto, Japan), including RT and real-time PCR. RT reactions were performed using a single miRNA-specific stem-loop RT primer as described by *Chen et al.*^44, 45^ (Supplementary Table1).

### Prediction of miRNA targets

The following bioinformatic algorithms were used to predict miRNA target sites: Target Scan6.1 (http://targetscan.org), Diana microT 4.0 (http://diana.cslab.ece.ntua.gr/DianaTools/index.php?r=microtv4/index), and MICRORNA.ORG (http://www.microrna.org/microrna/home.do). Targets overlapping among these three algorithms were selected as candidates for further analysis.

### Luciferase reporter and vector production

The *SPRY4* 3′-UTR luciferase reporter was constructed as follows: the full-length 3′-UTR of *SPRY4* (947 nucleotides) was amplified using cDNA from RD cells (forward: 5′-AGAAGCCTGTTTCTCCGTACA-3′, reverse: 5′-TCAGAAAGGCTTGTCGGG-3′). The primers were digested and cloned into the *Xba*1 site of pGL3 (Promega, Madison, WI, USA), checked for orientation, sequenced, and named Wt. Mutagenesis within selected seeding-sequence regions was conducted according to the following rules: G was changed to C and vice versa (Mut) (Figure 3b). Reporter assays were performed using the dual-luciferase assay system (Promega) and the luciferase signal was read using a TD-20/20 Luminometer (Turner Biosystems, Sunnyvale, CA, USA). PKC*α* Wt cDNAs were subcloned into a cytomegalovirus expression vector pRc/CMA (Invitrogen). Site-directed mutagenesis of PKC*α*-CDA was carried out using a Transformer Mutagenesis Kit (Clontech, Palo Alto, CA, USA). The primers used for Wt PKC*α*-coding and dominant-negative control PKC*α* were 5′-CGCAAAGGGGAGCTCAGGCAGAAGAAC-3′ and 5′-GTACGCCATCAGAATTCTGAAGAAGG-3′, as described previously.^46^ p38MAPK was activated in RD and RH30 RMS cells by transfection with hemagglutinin-tagged MKK6EE protein-expressing pcDNA3, as described previously.^47^ Briefly, MKK6EE chimera cDNAs were generated by swapping the region encoding the 77 N-terminal amino acid residues of MKK6EE using an internal *Bam*HI site and insertion into the pcDNA3 vector (Invitrogen) at *Hind*III/*Xba*I sites. For the c-Jun-Luc and Elk-1-Luc reporting system (Agilent Technologies, Palo Alto, CA, USA), cells were co-transfected with 0.1 *μ*g of pFA-c-Jun or pFA-Elk-1 activator plasmid (expressing proteins containing the DNA-binding domain of yeast GAL4 and activation domains of c-Jun and Elk-1), and 1 *μ*g of pFR-Luc plasmid (containing a synthetic promoter). The luciferase activity reflects the activation of a specific kinase.

### Oligonucleotide synthesis and transfection

LNA/DNA hybrid sense oligonucleotides (miR mimics) and antisense oligonucleotides (miR inhibitors) were chemically synthesized (Shanghai GeneCore Biotechnologies Co., Ltd, Shanghai, China). The sequences of the miRNA mimics and inhibitors are given in Supplementary Table S2. For transfection, 100 pmol oligonucleotides in 200 *μ*l of serum-free medium were mixed with 5 *μ*l of Lipofectamine 2000 transfection reagent (Invitrogen) dissolved in 200 *μ*l of the same medium. *SPRY4*-specific siRNAs were designed using BLOCK-iT RNAi Designer (Invitrogen). After confirmation by immunoblotting (Supplementary Figure S6), optimal sense (5'-GCAGUUCCUAUUGUAUAUATT-3') and antisense (5'-UAUAUACAAUAGGAACUGCTT-3') sequences were selected for *SPRY4* RNA interference (synthesized by Shanghai GeneCore Biotechnologies Co., Ltd). siRNA transfection was conducted following the same procedures used for miR at a final concentration of 30 nM.

### Immunohistochemistry and immunofluorescence staining

After blocking nonspecific binding sites with 10% normal rabbit serum, tissue sections were incubated with antibodies at 4 °C overnight. The slides were then incubated with peroxidase-conjugated IgG (Shanghai Genomics, Shanghai, China), stained with diaminobenzidine–chromogen substrate mixture (Dako, Santa Barbara, CA, USA), and counterstained with hematoxylin. Protein expression was classified according to the total IRS as described previously.^17^ Three independent pathologists blindly reviewed each stained slide and semiquantitatively categorized the intensity of staining. Briefly, staining extent was categorized as 0 (no positive cells), 1 (≤25% positive cells), 2 (>25% and ≤50% positive cells), or 3(>50% positive cells), and staining intensity was categorized as 0 (negative), 1 (weak), 2 (moderate), or 3 (strong). The individual categories were added to give a total IRS. IRS 6–9 and IRS 0–4 were defined as high expression and low expression of proteins, respectively.

The coverslips were placed at the bottom of each well of a multi-well plate and cultivated for 24 h in DMEM containing 10% fetal calf serum. After transfection, the coverslips were washed with ice-cold phosphate-buffered saline (PBS) and fixed with 4% paraformaldehyde for 20 min. The treated cells were permeabilized with 0.1% Triton X-100 (Beyotime, Shanghai, China) for 20 min. Expression levels of caspase-3, MHC, and myosin were analyzed using rabbit anti-cleaved caspase-3 (Beyotime, Shanghai, China), anti-MHC (Boster, Wuhan, China), and anti-myosin (Boster) in PBS containing 1% BSA. At least 200 cells/slide in random fields were counted under a fluorescence microscope (BX2-FLB3-000; Olympus, Tokyo, Japan) to determine the percentage of fluorescein isothiocyanate-positive cells.

### Cell growth assays

RMS cell lines were plated at 5 × 10^4^ cells per well in 96-well plates with transfection solutions containing miR-411-5p-M or miR-411-5p-I for 6 h. The culture medium was then replaced with differentiation medium. MTT and [^3^H]thymidine incorporation were explored after each treatment for 6, 12, 24, and 48 h. During the last 4 h of each treatment, cells were pulsed with MTT (Sigma, St Louis, MO, USA) 10 *μ*l/well or 3.7 × 10^4^ [^3^H]thymidine (1 *μ*Ci)/well. For the MTT assay, cells were incubated at 37 °C for 4 h to allow MTT formazan formation, and the medium and MTT were then replaced by 100 *μ*l dimethyl sulfoxide to dissolve the formazan crystals. The OD at 557 nm was determined using a microplate reader (Model 550; Bio-Rad, Hercules, CA, USA). The cells were pulsed with [^3^H]thymidine and rinsed with PBS, and DNA was precipitated with 10% (wt/vol) trichloroacetic acid for 30 min at 4 °C. The amount of [^3^H]thymidine incorporated was measured with a liquid scintillation counter.

### Cell cycle analysis

After co-transfection of MKK6EE with miR-411-5p-M, RD cells were collected at 6 and 48 h, fixed with 70% ice-cold ethanol, and stored at 4 °C for 2 h. The cells were centrifuged at 1000 r.p.m. for 10 min. Subsequently, the cell pellet was gently resuspended in 1 ml of hypotonic fluorochrome solution and then analyzed using FACScan flow cytometer (Coulter Epics Elite ESP, Miami, FL, USA).

### *In vivo* tumorigenicity

A total of 60 male BALB/c nude mice (5–6 weeks old, mean body weight 18±2 g) (SLAC Laboratory Animal Co. Ltd, Shanghai, China) were randomly divided into 3 groups of 20 mice each. For the tumorigenicity model, all mice were injected subcutaneously with 100 *μ*l RD cell suspensions (1 × 10^7^/l). For intratumoral transfection, 100 pmol miRNA mimics or inhibitors and negative control oligonucleotides in 100 *μ*l of PBS were mixed with 5 *μ*l of Lipofectamine 2000 transfection reagent (Invitrogen) dissolved in 100 *μ*l of the same PBS. Once the tumors reached ~0.5 cm^3^, 200 *μ*l of the above resulting transfection solutions were injected into the tumor twice weekly for 4 weeks. Tumor size was measured weekly in a blinded manner using calipers and tumor volume (*V*, mm^3^) was calculated as (*L* × *W*^2^)/2, where *L* is length (mm) and *W* is width (mm). All animal procedures were approved by the Ethics Review Committee for Animal Experimentation of Soochow University.

### Statistical analysis

Statistical analyses were performed using SPSS, version 20 (SPSS Inc., Chicago, IL, USA). Unless otherwise stated, error bars indicate S.E. and *P*-values <0.05 after two-tailed *t*-tests are denoted by an asterisk in the figures.

## Figures and Tables

**Table 1:** Correlations between TGF-*β*1, SPRY4, and P-p38 expression and clinical pathology

**Groups**	**No.**	**TGF-*β*1**			**SPRY4**			**P-p38**		
		**Low (0–4)**	**High (6–9)**	***P*-value**	**Low (0–4)**	**High (6–9)**	***P*-value**	**Low (0–4)**	**High (6–9)**	***P*-value**
RMS	72									
										
*Histological subtypes*										
ERMS	48	13 (27.1)	35 (72.9)	>0.05	10 (20.8)	38 (79.2)	>0.05	36 (75.0)	12 (25.0)	>0.05
ARMS	18	6 (33.3)	12 (66.7)		7 (38.9)	11 (61.1)		13 (72.2)	5 (27.8)	
PRMS	6	1 (16.7)	5 (83.3)		1 (16.7)	5 (83.3)		5 (83.3)	1 (16.7)	
										
*Grades*										
I	27	16 (59.3)	11 (40.7)		14 (51.9)	13 (48.1)		18 (66.7)	9 (33.3)	
II	23	5 (21.7)	18 (78.3)	*0.004*	7 (30.4)	16 (69.6)	*0.071*	18 (78.3)	5 (21.7)	*0.117*
III	22	2 (9.1)	20 (90.1)	*0.002*	4 (18.2)	18 (81.8)	*0.015*	20 (90.1)	2 (9.1)	*0.007*
										
*Prognosis*										
Recurrence free survival	41	26 (63.4)	15 (36.6)		24 (58.5)	17 (41.5)		31 (75.6)	10 (24.4)	
Relapse or metastasis	31	7 (22.6)	24 (77.4)	*0.008*	5 (16.1)	26 (83.9)	*0.007*	28 (90.3)	3 (9.7)	*0.019*

Abbreviations: ARMS, alveolar RMS; ERMS, embryonal RMS; IRS, immunocreative score; P-p38, phosphorylated p38; PRMS, pleomorphic RMS; RMS, rhabdomyosarcoma; SPRY4, sprouty homolog 4; TGF-*β*1, transforming growth factor-*β*1

All samples were confirmed histologically and classified as different grades according to the most differentiated areas in the tissue. Protein expression was classified according to grading systems, as mentioned in Materials and Methods, with a total IRS. IRS 6–9 and IRS 0–4 were defined as high expression and low expression of proteins, respectively. The table shows only the statistics results of high IRS of TGF-*β*1, SPRY4, and P-p38 among subtypes, grades and prognosis of RMS. The significant differences (*P*<0.05) can be found in different grades of RMS (II and III compared to I) and RMS with relapse/metastasis or not

## Abbreviations

RMS: rhabdomyosarcoma
miRNA: microRNA
TGF-*β*1: transforming growth factor-*β*1
SPRY4: sprouty homolog 4
MAPK: mitogen activated protein kinase
PKC: protein kinase C
MKK6EE: MAPK kinase 6
P-p38MAPK: phosphorylated p-38
ERK2: extracellular signal-regulated kinase 2
JNK: Jun N-terminal kinase
ARMS: alveolar RMS
ERMS: embryonal RMS
PRMS: pleomorphic RMS
MHC: myosin heavy chain
IRS: immunoreactive score
