# Supplementary material for: Autoregulatory loop between TGF-β1/miR-411-5p/SPRY4 and MAPK pathway in rhabdomyosarcoma modulates proliferation and differentiation
Source: Cell Death Dis. 2015 Aug 20;6(8):e1859–. doi: 10.1038/cddis.2015.225 (PMC4558514; doi:10.1038/cddis.2015.225)
Supplement: Supplementary Figure 1 [file cddis2015225x1.docx]

|  |  |
| --- | --- |
|  |  |
|  | |

**Supplementary Figure 1 .** **Validation of microarray analyses identified TGF-β1-suppressed miRNAs with Real-time RT-PCR assay.**
