# Supplementary material for: Autoregulatory loop between TGF-β1/miR-411-5p/SPRY4 and MAPK pathway in rhabdomyosarcoma modulates proliferation and differentiation
Source: Cell Death Dis. 2015 Aug 20;6(8):e1859–. doi: 10.1038/cddis.2015.225 (PMC4558514; doi:10.1038/cddis.2015.225)
Supplement: Supplementary Figure 2 [file cddis2015225x2.docx]

|  |
| --- |

**Supplementary Figure 2. Effect of miR-411-5p on proliferation of RMS cell line SJCRH30 *in vitro***
