# Supplementary figures and images for: Autoregulatory loop between TGF-β1/miR-411-5p/SPRY4 and MAPK pathway in rhabdomyosarcoma modulates proliferation and differentiation
Source: Cell Death Dis. 2015 Aug 20;6(8):e1859–. doi: 10.1038/cddis.2015.225 (PMC4558514; doi:10.1038/cddis.2015.225)

| **A** |
| --- |
| **B**  **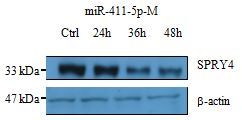** |

**Supplementary Figure 3 . Validation of SPRY4 as the target of miR-411-5p in SJCRH30 cell line.**

Supplement: Supplementary Figure 3 [file cddis2015225x3.docx]

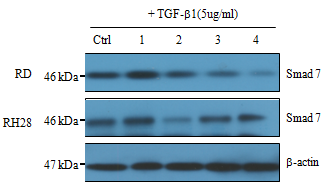


**Supplemental Figure 5. Validation of TGF-β1 gene knock-down.**

Supplement: Supplementary Figure 5 [file cddis2015225x5.docx]
