# Supplementary material for: Autoregulatory loop between TGF-β1/miR-411-5p/SPRY4 and MAPK pathway in rhabdomyosarcoma modulates proliferation and differentiation
Source: Cell Death Dis. 2015 Aug 20;6(8):e1859–. doi: 10.1038/cddis.2015.225 (PMC4558514; doi:10.1038/cddis.2015.225)
Supplement: Supplementary Figure 6 [file cddis2015225x6.docx]

| A   \| ID \| Sense(5' to 3') \| Antisense(5' to 3') \| \| --- \| --- \| --- \| \| SPRY4-567 \| 5'-CACCAUUGGAUCUCCUUCATT-3' \| 5'-UGAAGGAGAUCCAAUGGUGTT-3' \| \| SPRY4-1772 \| 5'-GCAGUUCCUAUUGUAUAUATT-3' \| 5'-UAUAUACAAUAGGAACUGCTT-3' \| \| SPRY4-3428 \| 5'-GGAGAGUCGAUUUACAUAATT-3' \| 5'-UUAUGUAAAUCGACUCUCCTT-3' \| \| Negative control \| 5'-UUCUCCGAACGUGUCACGUTT-3' \| 5'-ACGUGACACGUUCGGAGAATT-3' \| |
| --- | --- | --- | --- | --- | --- | --- | --- | --- | --- | --- | --- | --- | --- | --- | --- |
| B  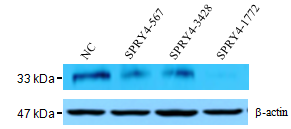 |

**Supplemental Figure 6. Validation of optimal sequence for *SPRY4* RNA interference.**
