# Supplementary material for: Autoregulatory loop between TGF-β1/miR-411-5p/SPRY4 and MAPK pathway in rhabdomyosarcoma modulates proliferation and differentiation
Source: Cell Death Dis. 2015 Aug 20;6(8):e1859–. doi: 10.1038/cddis.2015.225 (PMC4558514; doi:10.1038/cddis.2015.225)
Supplement: Supplementary Table 1 [file cddis2015225x7.docx]

**Supplemental Table 1. Primers used for real-time quantitative PCR**

| miRNA | Accession* | Primer sequences (5’→3’) |
| --- | --- | --- |
| hsa-miR-4275  hsa-miR-411-5p  hsa-miR-411-3p    hsa-miR-493-5p    hsa-miR-493-3p    hsa-miR-450b-5p  hsa-miR-4298    hsa-miR-2113  General downstream primer | MIMAT0016905  MIMAT0003329  MIMAT0004813  MIMAT0002813  MIMAT0003161  MIMAT0004909  MIMAT0016920  MIMAT0009206 | RT: CTCAACTGGTGTCGTGGAGTCGGCAATTCAGTTGAGAAAGAAGT  F: ACACTCCAGCTGGG CCAATTACCAC  RT: CTCAACTGGTGTCGTGGAGTCGGCAATTCAGTTGAGCGTACGCT  F: ACACTCCAGCTGGG TAGTAGACCGTATAG  RT: CTCAACTGGTGTCGTGGAGTCGGCAATTCAGTTGAGGGTTAGTG  F: ACACTCCAGCTGGG TATGTAACACGGTCCA  RT: CTCAACTGGTGTCGTGGAGTCGGCAATTCAGTTGAGAATGAAAG  F: ACACTCCAGCTGGG TTGTACATGGTAGGCT  RT: CTCAACTGGTGTCGTGGAGTCGGCAATTCAGTTGAGCCTGGCAC  F: ACACTCCAGCTGGG TGAAGGTCTACTGTGT  RT: CTCAACTGGTGTCGTGGAGTCGGCAATTCAGTTGAGTATTCAGG  F: ACACTCCAGCTGGG TTTTGCAATATGTTCC  RT: 5，-CTCAACTGGTGTCGTGGAGTCGGCAATTCAGTTGAGTGATAGCC  F：ACACTCCAGCTGGGGCATTGTGCAGGG  RT: CTCAACTGGTGTCGTGGAGTCGGCAATTCAGTTGAGGTGACAGA  F: ACACTCCAGCTGGG ATTTGTGCTTGGCTC  R: TGGTGTCGTGGAGTCG |

*<http://www.microrna.org/microrna/home.do>; F, forward primer; R, reverse primer
