# Supplementary material for: Autoregulatory loop between TGF-β1/miR-411-5p/SPRY4 and MAPK pathway in rhabdomyosarcoma modulates proliferation and differentiation
Source: Cell Death Dis. 2015 Aug 20;6(8):e1859–. doi: 10.1038/cddis.2015.225 (PMC4558514; doi:10.1038/cddis.2015.225)
Supplement: Supplementary Table 2 [file cddis2015225x8.docx]

**Supplementary Table. 2. Mimics and inhibitors of miRNAs used in this study**

| miRNA | Accession* | Mimics sequences (5’→3’) | Inhibitors sequences (5’→3’) |
| --- | --- | --- | --- |
| hsa-miR-4275  hsa-miR-411-5p  hsa-miR-411-3p  hsa-miR-493-5p  hsa-miR-493-3p  hsa-miR-450b-5p | MIMAT0016905  MIMAT0003329  MIMAT0004813  MIMAT0002813  MIMAT0003161  MIMAT0004909 | CCAAUUACCACUUCUUU  UAGUAGACCGUAUAGCGUACG  UAUGUAACACGGUCCACUAACC  UUGUACAUGGUAGGCUUUCAUU  UGAAGGUCUACUGUGUGCCAGG  UUUUGCAAUAUGUUCCUGAAUA | AAAGAAGUGGUAAUUGG  CGUACGCUAUACGGUCUACUA  GGUUAGUGGACCGUGUUACAUA  AAUGAAAGCCUACCAUGUACAA  CCUGGCACACAGUAGACCUUCA  UAUUCAGGAACAUAUUGCAAAA |

*<http://www.microrna.org/microrna/home.do>
